# Supplementary material for: Quantifying post-transcriptional regulation in the development of Drosophila melanogaster
Source: Nat Commun. 2018 Nov 26;9:4970. doi: 10.1038/s41467-018-07455-9 (PMC6255845; doi:10.1038/s41467-018-07455-9)
Supplement: Supplementary file 1 — Supplementary Information [file 41467_2018_7455_MOESM1_ESM.pdf]

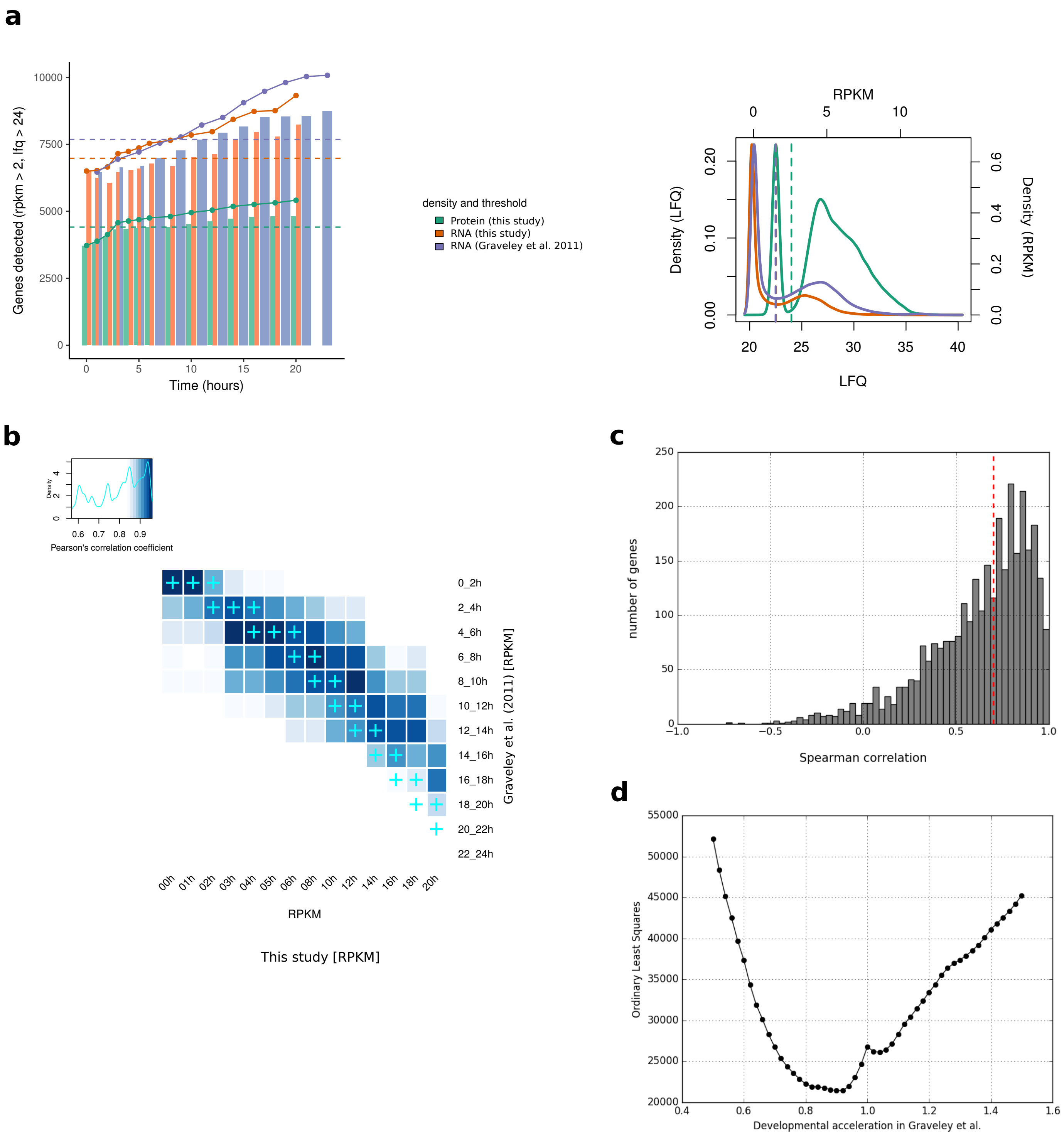

Supplementary Figure 1 - Comparison of *Drosophila* embryogenesis RNA-Seq datasets: a Coverage of transcriptomic and proteomic data. Left panel: Number of genes detected in each sample for RNA-Seq from Graveley et al. (2011) (purple), RNA-Seq in this study (orange) and mass-spectrometry based proteomics (green). Right panel: Density distribution of transcript abundance in Reads per kilo base per million mapped reads (RPKM) from this study and Graveley et al. (2011) as well as density distribution of log2 measured Label-Free Quantification (LFQ). Same color code as in the left panel. **b** Global correlation between samples of RNA-Seq data from Graveley et al. (2011) and RNA-Seq in this study. Pearson correlations between the two transcriptomes for each embryogenesis time point based on commonly identified genes in both datasets (12,478 genes). Approximately matching time points between studies are indicated by (+). **c** Individual mRNAs show similar dynamics in Graveley et al. (2011) and our dataset. For each of the 12,478 common genes, the Spearman correlation between time courses were calculated along the time course (in case of non-matching time points, our data was compared to the earlier time point in the published data). The histogram shows the distribution of correlation coefficients over all 12,478 genes. The red dashed line indicates the median of all Spearman correlations. **d** Developmental progress was accelerated in the Graveley et al. (2011) dataset compared to our experiment. The time axis for Graveley et al. (2011) was compressed by an acceleration factor (x-axis; 1: no acceleration; <1: acceleration; >1: deceleration of mRNA dynamics). Differences in the mRNA time courses of both datasets were quantified by calculating the ordinary least squares difference of summed transcript abundance of all time points and genes (y-axis). Data from Graveley et al. (2011) (multiplied by the acceleration factor) underwent linear interpolation to obtain matching time points with our RNA-Seq dataset.

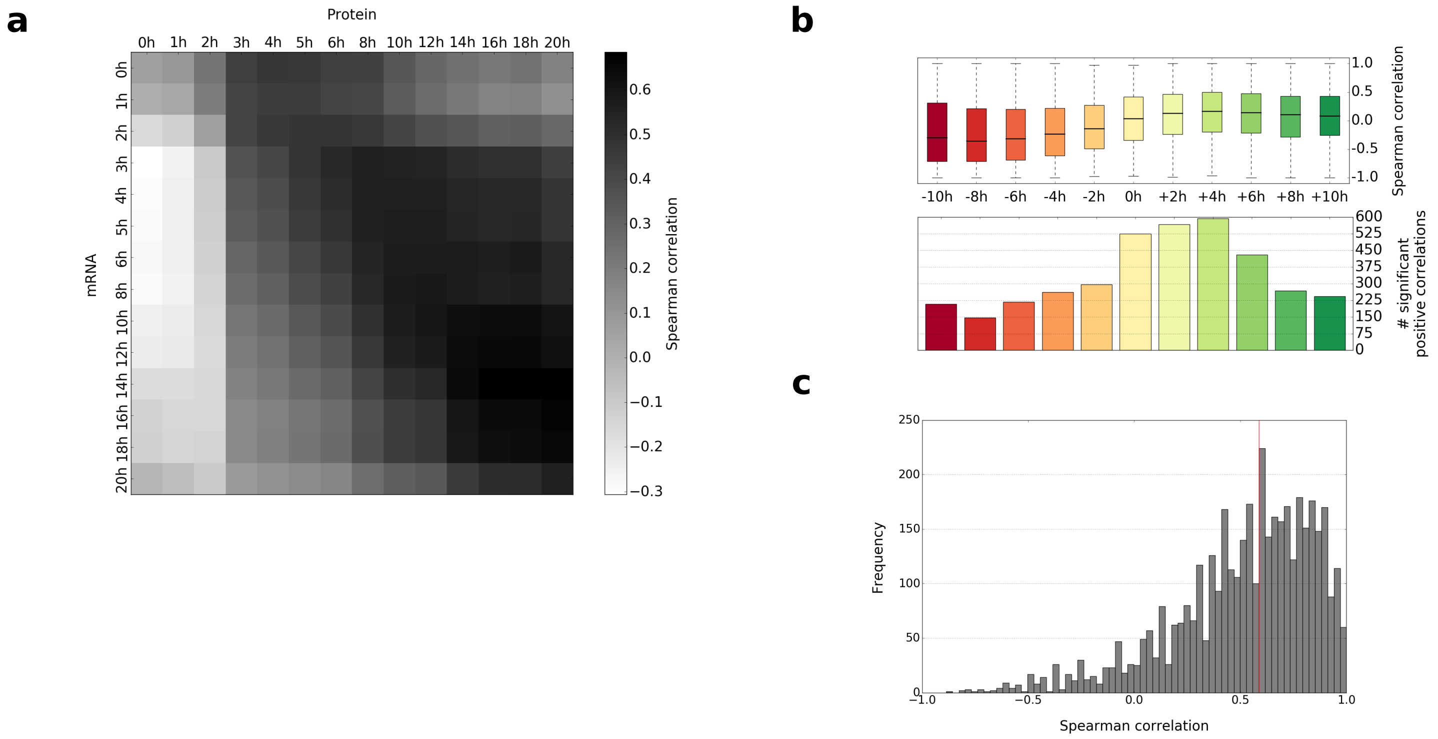

Supplementary Figure 2 - Minor impact of proteome quantification method (LFQ vs. iBAQ) on mRNA/protein correlation: a Heatmap showing the global mRNA/protein correlation using iBAQ quantification for proteome measurements. The plot shows the same as Figure 2A, except that for the proteome data iBAQ instead of LFQ quantification was used. **b** Local correlation of mRNA and protein time courses (similar to Figures 2C and D). The upper panel shows the distribution of calculated Spearman correlation for different time shifts where mRNA is shifted relative to iBAQ quantified protein (line: median; boxes: quartiles; whiskers: 95-percentile) (positive values correspond to a mRNA delay). Number of significant positive correlations ($p < 0.05$, $\rho > 0.0$) are indicated in the second panel. **c** Histogram of the distribution of maximum mRNA/protein correlations if only positive time-shifts are considered. Protein abundance was based on iBAQ values. The red line indicates the median of all Spearman correlations.

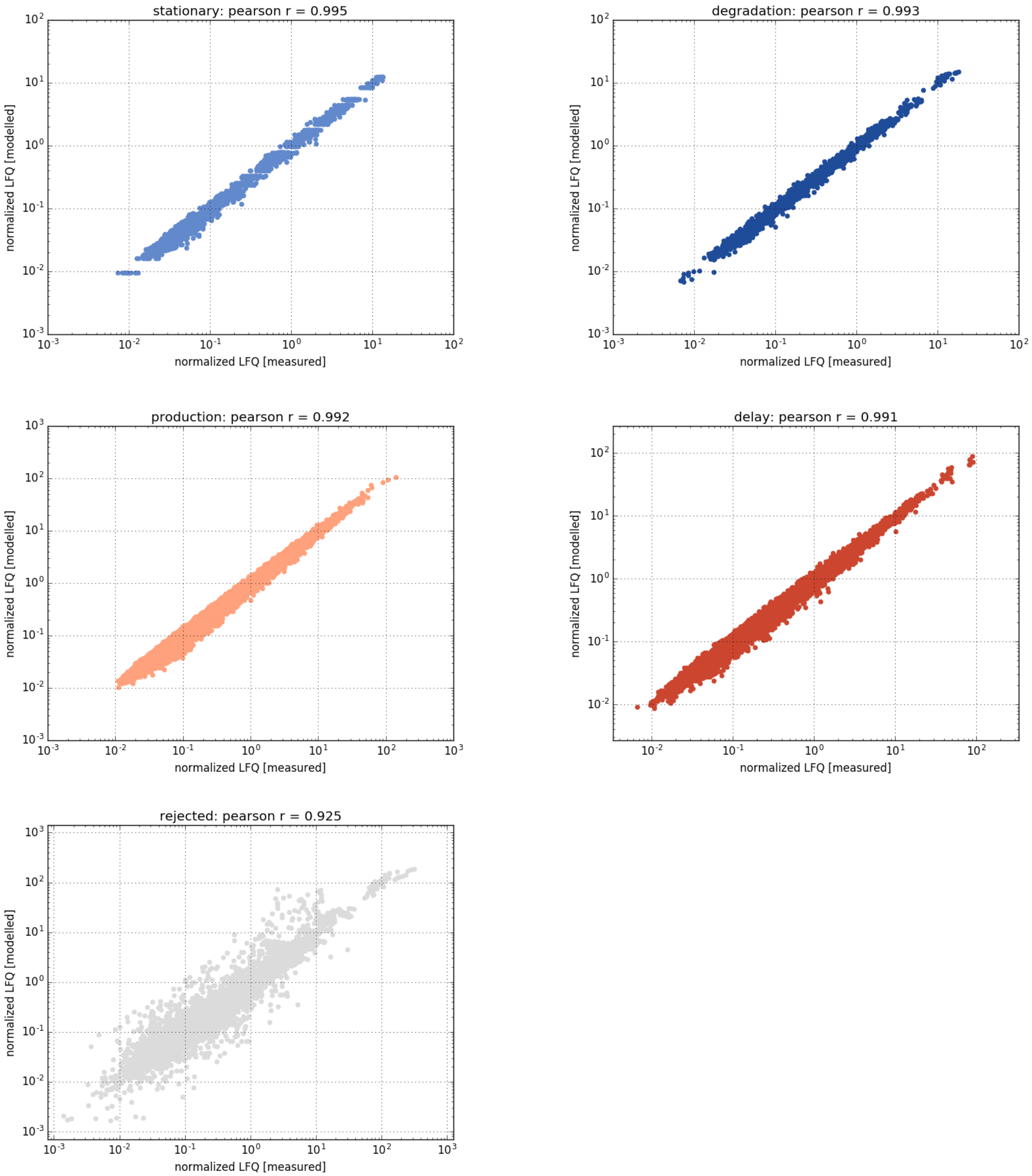

Supplementary Figure 3 - Good agreement between measured and modelled protein expression values: Scatter plots showing Pearson correlation between measured protein expression values [log2 LFQ] and simulated protein expression abundances returned by the best-fit model across all time points and genes. Since for potentially post-transcriptionally regulated proteins all models are rejected, respective modeled protein expression resulting from the best fit in the delay model is shown, as this model contains most-degrees of freedom and contains all other model variants.

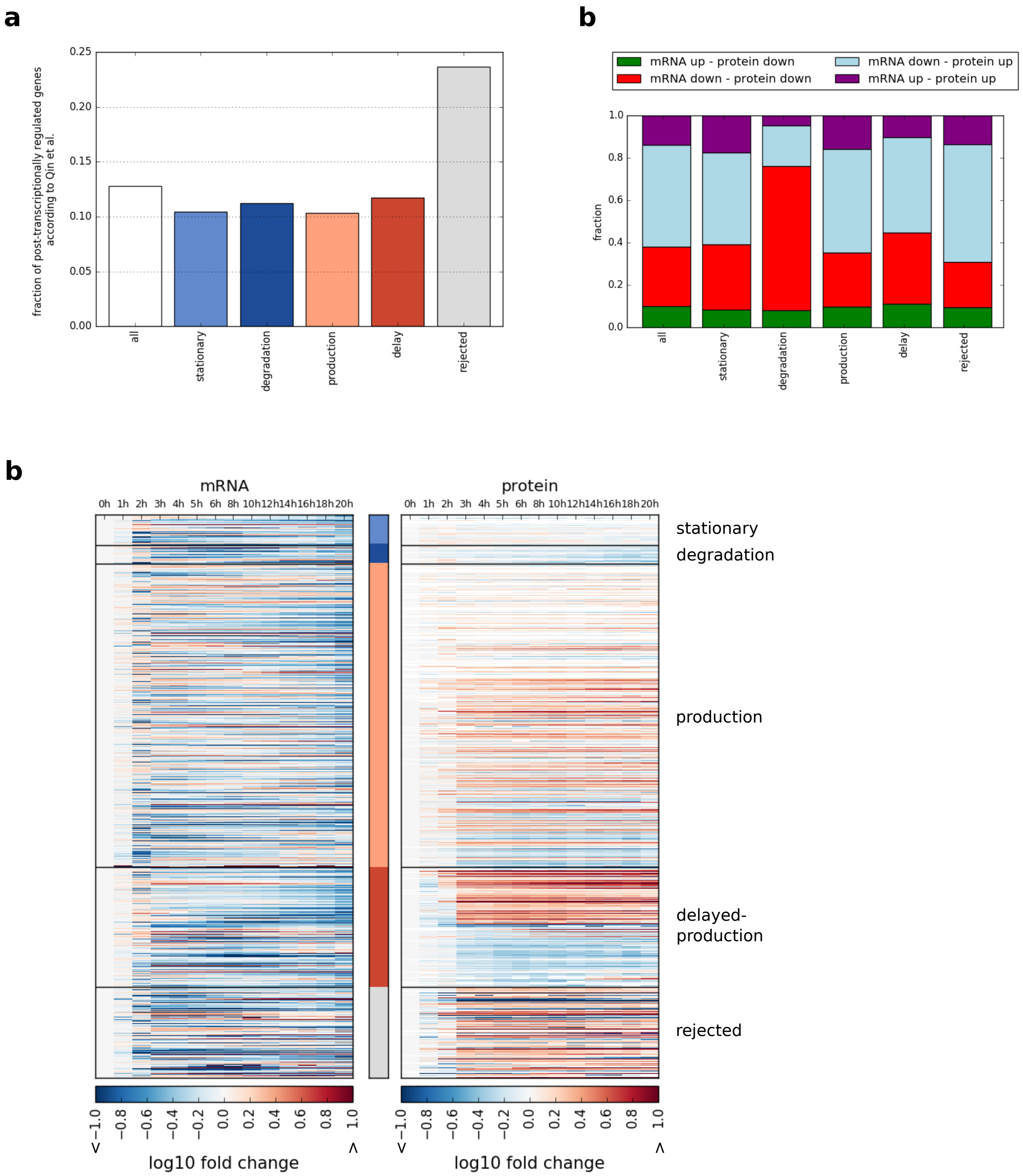

Supplementary Figure 4 - Protein classes show distinct mRNA-protein dynamics: a Genes previously identified as post-transcriptionally regulated during embryonic development by ribosome profiling (Qin et al. (2007)) are enriched among mRNA/protein pairs assigned to the rejected group. Based on the results by Qin et al. (2007), we derived a list of 1,522 post-transcriptionally regulated genes by considering the union of individual lists provided in the supplement of the mentioned study. **b** mRNA-protein dynamics across different classes of protein expression regulation. Stacked bar plots show the fraction of genes belonging to the four dynamic time course clusters (up-/downregulation of mRNA/protein), as indicated in Figure 1B. **c** Heatmap of mRNA and protein expression dynamics sorted according to the model-based classification. Within each class, time courses are sorted according to the estimated initial protein level (stationary), the degradation rate (degradation), the production rate (production) or the delay (delayed-production). Log10 fold-changes relative to $t=0$ are shown within the range -1 and 1.

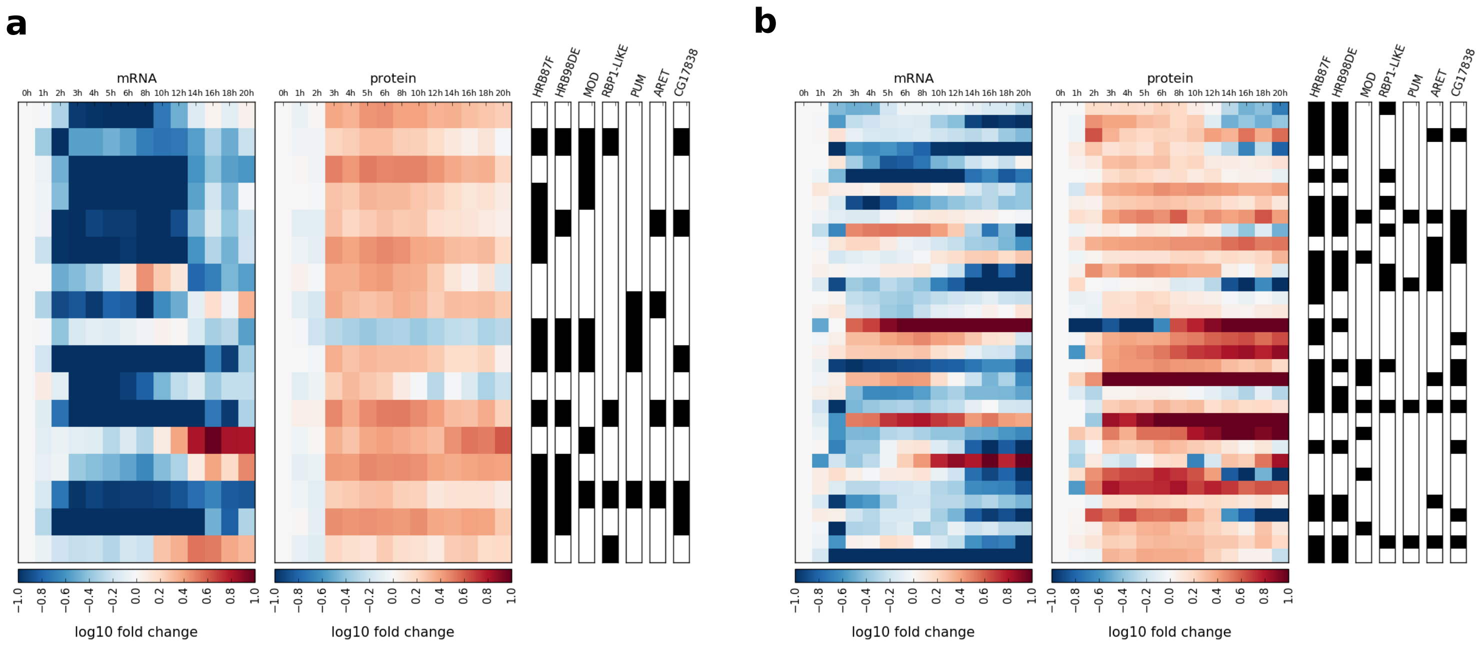

Supplementary Figure 5 - Temporal mRNA/protein dynamics of genes involved in sugar metabolism and cell cycle: Heatmaps of mRNA and protein expression for genes with GO-terms associated with (**a**) glucose metabolism (**b**) and cell cycle that were rejected based on model fitting. Shown are log10 fold-changes (relative to t=0h) of (**a**) 17 genes for sugar metabolism and (**b**) 34 genes for cell-cycle regulation and mitosis. For specific GO-Terms refer to Methods. The columns at the right of each figure depict presence of the binding motif for the indicated RBP within the longest coding sequence of the transcript.

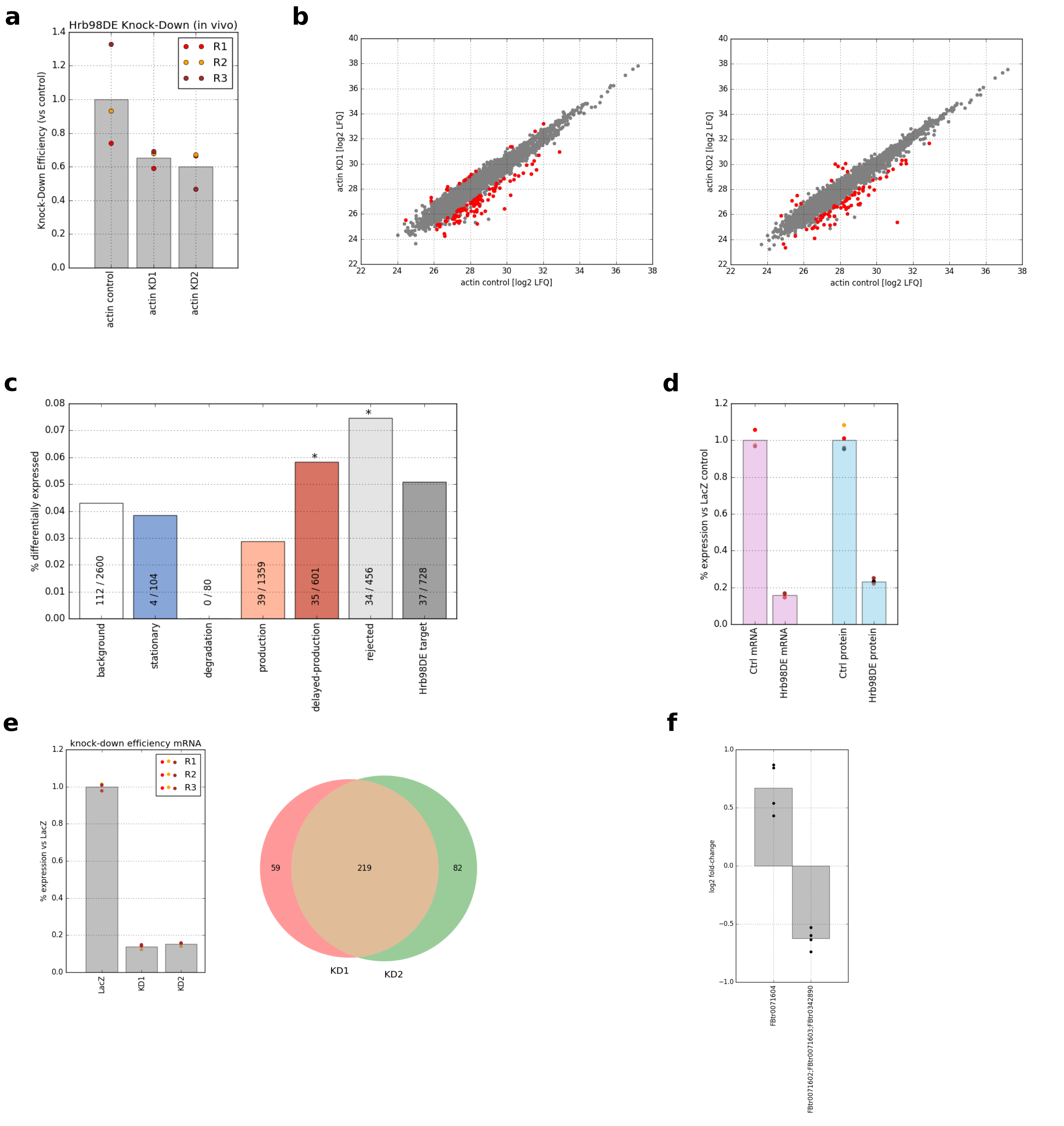

Supplementary Figure 6 - Hrb98DE knock-down studies during in vivo Drosophila development and in S2R+ cells: a Efficiency of in vivo Hrb98DE knock-down at the protein level 7:45h after egg deposition (29°C) as assessed by mass spectrometry. Two distinct dsRNAs were expressed using an actin-GAL4 driver (KD1 and 2) and each experiment was performed in triplicates (circles). Hrb98DE protein expression in knockdown animals remained at ~50% of the control line, illustrating a moderate knockdown efficiency. **b** Scatter plots showing global in vivo proteome changes in Hrb98DE KD1 (left) and KD2 (right) lines. Red dots highlight significantly changing proteins. **c** Bar graph (similar to Figure 7A) - Model-predicted post-transcriptionally regulated proteins are enriched for differentially expressed proteins (hypergeometric test, $p < 0.05$, indicated by *). **d** Hrb98DE knockdown efficiency in S2R+ cells. Shown are residual fractions of Hrb98DE mRNA (left, RNA-Seq) or protein (right, mass spectrometry) compared to LacZ knock-down control treated S2R+-cells 5 days after dsRNA treatment. **e** Knockdown with two independent Hrb98DE dsRNAs S2R+ cells shows highly similar effects at the mRNA level as assessed by RNA-seq: The left panel shows the knockdown efficiency of two distinct dsRNAs (KD1 and KD2), each performed in triplicates (R1-R3) shown as RNA expression level compared to LacZ control. The right panel shows a strong overlap of the sets of differentially expressed mRNAs (BH corrected p-value < 0.05, fold-change > 1.5) in the two knock-down experiments. **f** Isoform switch of domino (dom) on protein level upon Hrb98DDE knockdown in S2R+ cells. Barplots represent log2 fold expression-changes of two alternative domino protein groups (FBtr0071604 and FBtr0071602;FBtr0071603;FBtr0342890). Individual replicates are shown in black.

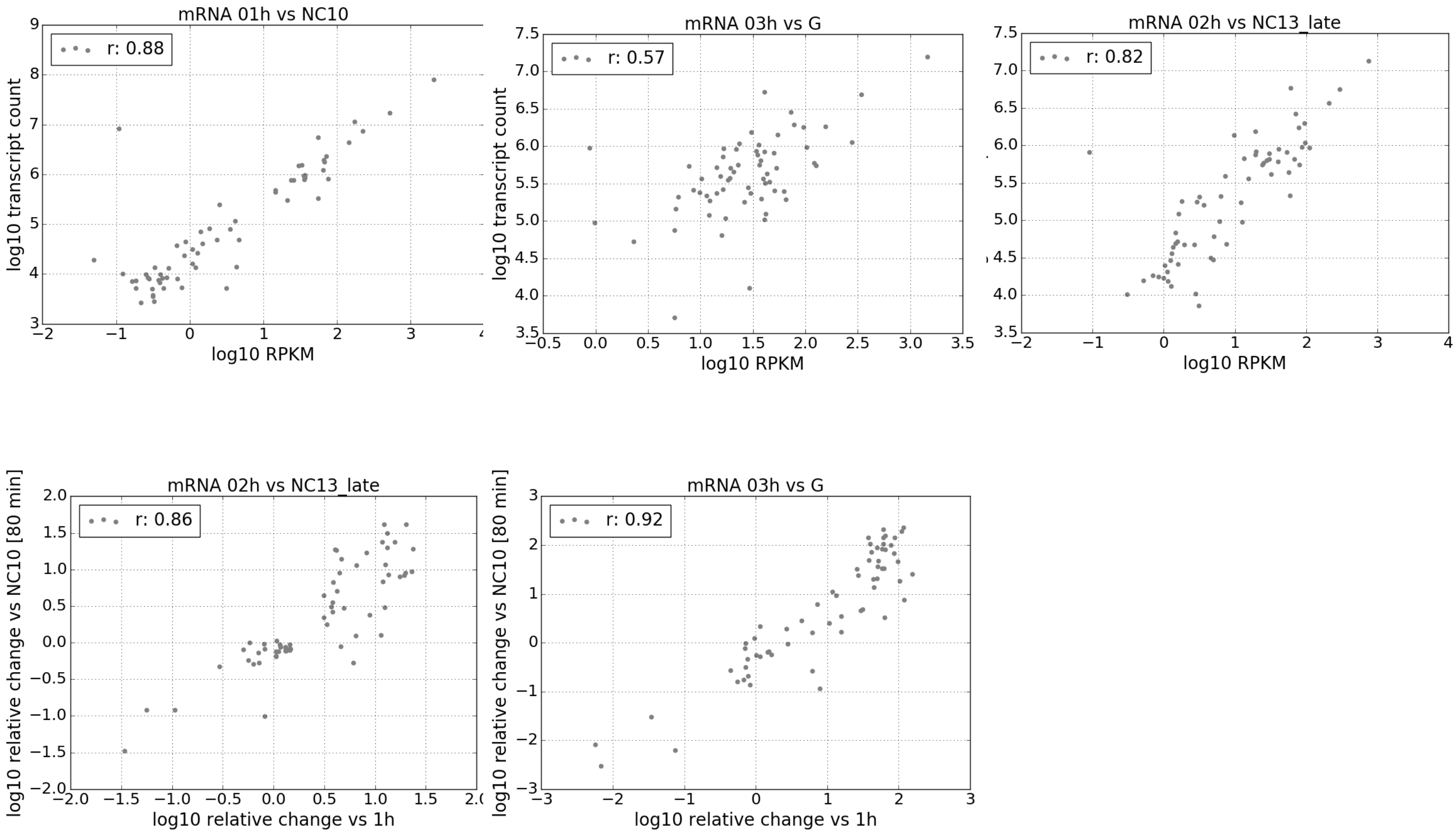

Supplementary Figure 7 - Per-sample normalization of mRNA expression levels into relative expression units agrees well with previous absolute mRNA quantifications: Comparison of our relative RNA measurements normalized to total RNA counts in each sample (RPKM, x-axis) with absolute RNA quantifications reported in Sandler & Stathopoulos (y-axis) of 64 genes overlapping between both studies. The top and bottom rows show comparisons of corresponding time points (NC10: nuclear cycle 10, G: gastrulation, NC13: nulcear cycle 13) and fold-changes between time points, respectively. The Pearson correlation coefficient (r) is indicated on the top left.

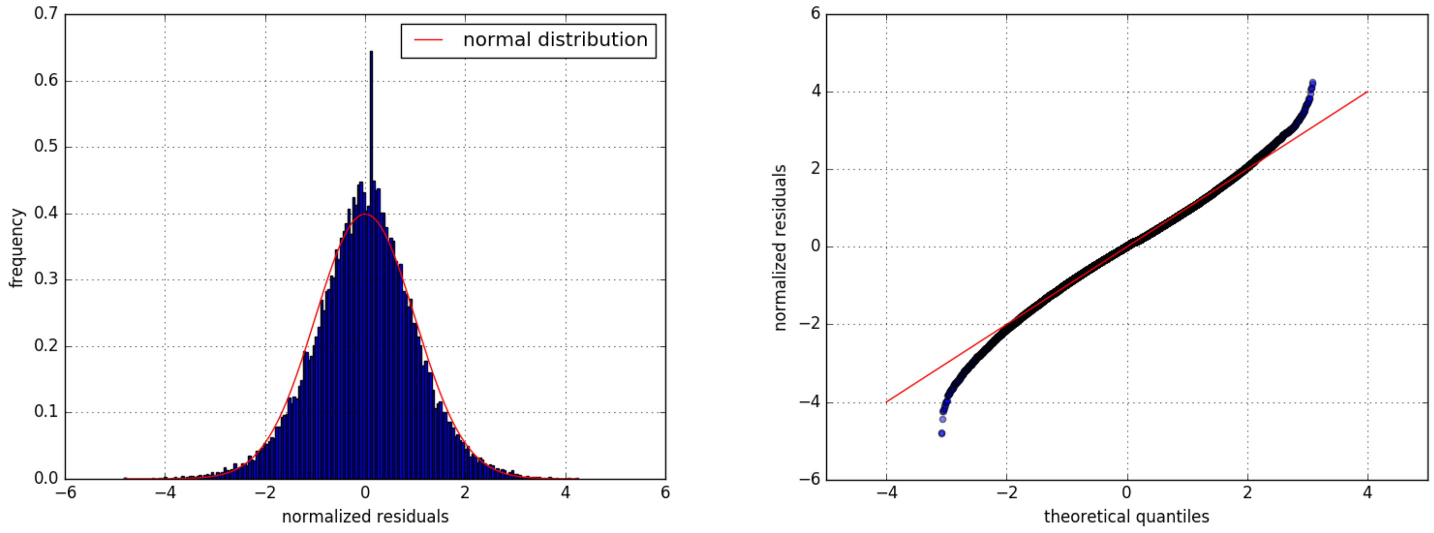

Supplementary Figure 8 - Analysis of model residuals shows good agreement with normal distribution: Histogram and Q-Q-Plot of residuals over all time points and all non-rejected proteins (*n=3153*) fitted by the delay model indicate a close-to-normal distribution. In the left plot normal distribution is shown in red, while the red line on the right indicates a perfect match between theoretical quantiles and normalized residuals.

Supplementary Table 1: Primer sequences

Supplementary Table 1: primer sequences to amplify the dsRNA template and qRT-PCR primer sequences

Hrb98DE dsRNA1 fwd	TAATACGACTCACTATAGATGGTGAACTCGAACCAGAACC
Hrb98DE dsRNA1 rev	TAATACGACTCACTATAGGTAGGGCTGCATGCGATTG
Hrb98DE dsRNA2 fwd	TAATACGACTCACTATAGGGACTACCGTACCACCGACGAG
Hrb98DE dsRNA2 rev	TAATACGACTCACTATAGCACCTCCCTGTTGGTCATTCTG
Hrb98DE qPCR fwd	CAAGGAGGTGGTGGATTCAAAG
Hrb98De qPCR rev	CAATATCTGCGGTTGTTGCCAC
rpl15 qPCR fwd	GCGCAATCCAATACGAGTTC
rpl15 qPCR rev	AGGATGCACTTATGGCAAGC

Supplementary Note 1: Comparison of our RNA-Seq data with Graveley et al. (2011)

Calculating the Spearman correlation between individual mRNA in our data and data by Graveley et al. (2011) [1] we chose the set of 3125 genes present in both datasets. Samples were paired according to the lower limit of the measurement interval.

Our transcriptome matches the published RNA-Seq dataset at similar stages of development when calculating pairwise correlations of all common reads between samples (Supplementary Figure 1B). Of note, the correlation of corresponding developmental time points tended to be higher early in development, and declined at later stages, hinting to a systematic deviation between both datasets. Indeed, late in development, the maximum correlation of our time points occurs with slightly earlier developmental time points of the previously published RNA-Seq data. This suggests that the embryonic development in the published dataset was accelerated when compared to our conditions.

To further test our hypothesis of altered developmental speed, we related the mRNA time courses for individual genes between both RNA-Seq datasets, and observed a strong correlation in the mRNA dynamics for the majority of genes (Supplementary Figure 1C). Time course expression of both mRNA time courses was normalized by its mean and standard deviation (z-score), in order to focus only on time course behavior and avoid issues resulting from differences in absolute gene expression. Data from Graveley et al. (2011) was defined at $t_{Graveley} = \{0, 2, 4, 6, 8, 10, 12, 14, 16, 18, 20, 22\}$, while our data was defined at $t_{Becker} = \{0, 1, 2, 3, 4, 5,\ 6, 8, 10, 12, 14, 16, 18, 20\}$. Expression data from Graveley et al. (2011) was compressed along its time axis by division of t with a constant factor chosen between 0.5 and 1.5. By linear interpolation of the compressed time course at time points corresponding to t_{Becker}, the ordinary least squares difference between our RNA-Seq data and compressed data from Graveley et al. (2011) was calculated over all genes and all time points. In line with faster developmental progress in Graveley et al. (2011), we could increase the overlap between both datasets by assuming a 8% acceleration (ca. 1.5h at the last measured time point) in our developmental progression (Supplementary Figure 1D).

Supplementary Note 2: Comparison of LFQ and iBAQ normalized protein values

Multiple approaches for the normalization and quantification of proteomics data exist. In particular, the use of LFQ ('label-free quantification' [2]) or iBAQ ('intensity-based absolute quantification' [3]) protein quantification remains an open question when analysing proteomics data. Here we show that the use of LFQ or iBAQ does not strongly affect the correlation between mRNA and protein.

Our filtering approach, in which only proteins measured with high accuracy (number of time-points > 10) were selected, yielded 3761 identified proteins in the case of using LFQ quantification, while iBAQ quantification yielded 4179 identified proteins.

When assessing between sample correlation over all genes (compare Figure 2A of the manuscript), iBAQ quantification yields a slightly higher correlation compared to LFQ quantification. The maximum correlation between two matching time-points in the case of iBAQ quantification was 0.64 compared to maximum correlation of 0.56 when using LFQ quantification (Supplementary Figure 2A). The maximum correlation between non-matching time-points calculated from iBAQ quantified protein was 0.68 (mRNA 12h - protein 16h) compared to maximum correlation of 0.63 based on LFQ values (mRNA 12h - protein 16h).

We however noticed a less pronounced impact of the quantification protocol on the local mRNA-protein correlation, in which we relate the RNA and protein time-courses for each gene separately (compare Figure 2C of the manuscript). The distribution of calculated pairwise spearman correlations remains rather broad over all mRNA/protein pairs, with a mean correlation of $\rho^{iBAQ} = 0.025$ and $\rho^{LFQ} = 0.009$, if no time-shift between mRNA and protein is introduced (Supplementary Figure 2B). Based on iBAQ values, the time-shift producing maximum mean mRNA/protein correlation ($\rho^{iBAQ} = 0.140$ is reached when a 4h time-lag of protein relative to mRNA is assumed. The corresponding maximum mean correlation is reached at 6h based on LFQ quantification ($\rho^{LFQ} = 0.158$). Further, the total number of significant positive correlation between mRNA/protein pairs for non-shifted and shifted time-courses is comparable for LFQ or iBAQ values. When considering only maximum correlation for positive time-shifts we observe a median correlation of $\rho^{iBAQ} = 0.59$ over all genes, which is comparable to $\rho^{LFQ} = 0.58$.

In conclusion, the choice of protein quantification by iBAQ or LFQ generally does not impact the observed low mRNA/protein correlation.

Supplementary Note 3: Model fitting and protein classification

In order to classify mRNA/protein measurements into groups we have designed 4 alternative models describing kinetics of mRNA translation. The simplest model assumes no change in protein abundance ('stationary'), while in a second model only protein degradation is assumed ('degradation'). In a third model, protein is degraded but also produced from its mRNA template ('production'). The most complex model again assumes protein production and degradation, however protein production is halted until a certain time after the beginning of the time-course ('delayed-production'). In the following, model formulations and their respective equations are provided.

Model formulations

Stationary model

In the simplest model, protein is assumed to be stationary, i.e. it is neither produced nor degraded over the time-course:

$$\frac{dy(t, y_0)}{dt} = 0, \tag{1}$$

with the initial condition

$$y(t) = y_0. \tag{2}$$

Here $y(t)$ denotes protein expression at time t and y_0 denotes protein expression at $t = 0$. When fitting this model to the data, we can explicitly calculate the only parameter of this model (y_0) as the weighted mean average over all given data-points:

$$y_0 = \frac{1}{n}\sum_{i=0}^{n}\frac{y_i}{\sigma_i^2} \tag{3}$$

In order to account for the variance in gene expression (σ) an error model is assumed, where the variance is estimated from the relation between mean expression values and their standard deviation. Fitting this linear error to the data a relative error of 17% was assigned to each data point.

Degradation model

In a more complex model, first-order protein degradation is added. Here the differential equation

$$\frac{dy(t, y_0, \lambda)}{dt} = -\lambda y(t) \tag{4}$$

with the initial condition

$$y_0 = y(t = 0) \tag{5}$$

can be solved explicitly as

$$y(t) = y_0 e^{-\lambda t}. \tag{6}$$

Again y_0 denotes protein expression at $t = 0$, while λ corresponds to the protein degradation rate.

Production model

The standard model of translational kinetics includes both linear protein degradation as well as protein production from mRNA. If mRNA concentration is given by linear interpolation $u(t) = mt + b$ between measured time-points, the differential equation for the production model is formulated as

$$\frac{dy(t, y_0, \alpha, \lambda)}{dt} = \alpha(mt + b) - \lambda y(t) \tag{7}$$

with the initial condition

$$y_0 = y(t = 0). \tag{8}$$

Here λ denotes the protein degradation rate, α the protein production rate and y_0 initial protein concentration. The explicit solution of Equation 18 is given as

$$y(t) = \alpha b \lambda^{-1} - \alpha m \lambda^{-2} + \alpha m t \lambda^{-1} + c e^{-\lambda t}. \tag{9}$$

In order to obtain the value of c we use the initial condition, at which point $t = 0$:

$$y_0 = \alpha b \lambda^{-1} - \alpha m \lambda^{-2} + c. \tag{10}$$

Equation 10 solved w.r.t c becomes

$$c = y_0 - \alpha b \lambda^{-1} + \alpha m \lambda^{-2}. \quad (11)$$

Please note that Equation 9 is solved on each measurement interval. Hence indices for m_k and b_k need to be introduced, with $t_k \in \{0, 1, 2, 3, 4, 5, 6, 8, 10, 12, 14, 16, 18\}$. The respective equations then reformulate to

$$y(t - t_k) = \alpha b_k \lambda^{-1} - \alpha m_k \lambda^{-2} + \alpha m_k (t - t_k) \lambda^{-1} + c e^{-\lambda(t - t_k)} \quad (12)$$

and

$$c_k = y_k - \alpha b_k \lambda^{-1} + \alpha m_k \lambda^{-2}, \quad (13)$$

where

$$y_k = y(t = t_k). \quad (14)$$

Delayed-production model

In a more elaborate model we assume a delay in protein production

$$\frac{dy(t, y_0, \alpha, \lambda, \tau)}{dt} = h(t - \tau)\alpha(mt + b) - \lambda y(t), \quad (15)$$

where τ corresponds to the time-delay until which protein translation is inhibited. In the equation above h denotes the Heaviside function. Please note that we solve the delay model on the given time-intervals $t_k \in \{\tau, 0, 1, 2, 3, 4, 5, 6, 8, 10, 12, 14, 16, 18\}$ by using the degradation model (Equation 6) if $t < \tau$ and the production model (Equation 9) if $t \geq \tau$.

Parameter estimation

The different model parameters $\theta = \{y_0, \lambda, \alpha, \tau\}$ are estimated by minimizing the weighted least squares distance between model and data:

$$\chi^2(\theta) = \sum_i^n \frac{(y_i^{model}(\theta) - y_i^{data})^2}{\sigma_i^2} \tag{16}$$

χ^2 is minimized using a multi-start local optimization procedure (scipy.optimize. least_squares). Each data-point is weighted according to its standard deviation σ_i, which has been estimated by fitting a linear error model to the protein expression data (17% relative noise). For each model a different number of initial parameter vectors is chosen via latin-hypercube sampling (degradation: 5, production: 10, delay: 25)

Classification of mRNA/protein pairs

After fitting models to the available data, the feasibility of each model is tested using a combination of χ^2- and Durbin-Watson test. The empirical distribution of both test-statistics is estimated by applying a parametric bootstrap strategy. Here, modelled protein expression resulting from the best model fit is resampled assuming normally distributed noise with mean zero and standard deviation according to the linear error model of 17% relative noise.

$$y^{bootstrap} = y^{model}(\hat{\theta}) + \mathcal{N}(0, \sigma_i) \tag{17}$$

Empirical distributions of test statistics are estimated based on 1000 bootstrap samples and p-values for each test statistic calculated using the empirical cumulative density function (statsmodels.distributions.empirical_distribution.ECDF). P-values for each test statistic are corrected using the Benjamini-Hochberg procedure (statsmodels.stats. multitest.multipletests). A threshold of BH corrected p-value < 0.05 for either of the considered test-statistics serves as a rejection criterion.

In case all models need to be rejected for a particular mRNA/protein pair, it is assumed to be post-transcriptionally regulated. In any other case, model selection between all non-rejected models is carried out using a step-wise likelihood ratio test. During the model selection process we select between different feasible models with varying numbers of parameters. The stationary and degradation model contain one and two parameters respectively, while the production and delay model contain three and four parameters. For a more complex model (i.e. a model with more parameters) to be preferred over a simpler one, it is necessary that the increase in the quality of the model fit of the more complex model compared to the simpler one exceeds a threshold given by the χ^2 test-statistic with n degrees of freedom, where n related to the difference in degrees of freedom between two tested model. This ensures that both model fit and number of parameters are balanced and over-fitting is prevented.

However, when comparing for example the degradation model with the production model from a biological point of view, it is at least questionable whether the degradation model is indeed the simpler one: In reality one would need to assume an additional factor blocking translation, meaning that the 'implicit' number of parameters should be larger

in the degradation model compared to the production model. We therefore introduce a set of correction criteria to account for ‘wrongly’ classified proteins:

- The protein is assigned to the stationary or degradation class
- The production model for this protein was not rejected based on the χ^2-test or Durbin-Watson test
- The estimated production rate in the production model is bigger than 2e-5

If all of the above criteria are met, a protein originally assigned to the stationary or degradation group is now re-assigned to the production model.

Supplementary Note 4: Benchmarking

Benchmark data

We evaluate our approach for the identification of post-transcriptionally regulated mRNA/ protein pairs based on their measured expression time-courses using benchmark data. In this *in silico* generated dataset the set of post-transcriptionally regulated genes is known. In order to obtain mRNA and protein expression data for genes following normal translation, we selected a random mRNA from the embryonic time-course and simulated protein expression dynamics using the production model:

$$\frac{dy(t, y_0, \alpha, \lambda)}{dt} = \alpha u(t) - \lambda y(t). \tag{18}$$

Initial protein level (y_0), degradation rate (λ), and production rate (α) were sampled from normal distributions fit to the estimated parameters of the original data.

As a second set, we simulated post-transcriptionally regulated mRNA/protein pairs by assuming net production of a protein to depend on the expression of a second, randomly chosen protein time-course from the embryo data-set. Expression measurements for the regulating protein were scaled between 0 and 1 (min/max) in order to ensure sufficient dynamic range. Finally expression of the regulated protein was simulated according to the following equation:

$$\frac{dy(t, y_0, \alpha, \lambda)}{dt} = \alpha \frac{p^h}{p^h + k} u(t) - \lambda y(t). \tag{19}$$

Here p corresponds to the gene expression of the regulating protein, while y describes expression of the regulated protein. Model parameter h, describing the sensitivity of the production rate with respect to the regulating protein was sampled from the interval $\pm\{1, 4\}$, while k was set to 0.5. All other model parameters parameters were sampled as above.

The presence of a regulating protein however does not imply a string impact on expression of the regulated protein. We therefore considered a protein to be post-transcriptionally regulated only if the weighted least-squares distance between gene expression obtained assuming post-transcriptional gene regulation and protein expression of the regular production model was above $\chi^2 > 22.362$. Finally, normally distributed noise was added to each data-point (5%, 10%, 15%, 20%, 25%). In each of the benchmark data-sets with different levels of experimental noise we simulated 100 gene expression time-courses, of which 300 are post-transcriptionally regulated.

Evaluation

In order to evaluate the performance of the classification approach we checked how well genes predicted to be post-transcriptionally regulated matched with the set of true post-transcriptionally regulated genes. Accordingly, we calculated precision (rate of correct prediction) and recall (rate of correctly recovered) values (Supplementary Table 2).

Supplementary Table 2: Performance evaluation Shown are performance indicators of the classification approach. ρ denotes the Pearsons correlation between true and estimated parameter values.

noise level	predicted	precision	$\text{precision}_{random}$	recall	recall_{random}	ρ_{y0}	$\rho_{production}$	$\rho_{degradation}$
0.05	167	0.91	0.30	0.51	0.17	1.00	0.74	0.93
0.10	100	0.92	0.30	0.31	0.10	1.00	0.70	0.87
0.15	78	0.96	0.30	0.25	0.08	1.00	0.58	0.79
0.20	81	0.94	0.30	0.25	0.08	1.00	0.61	0.79
0.25	67	0.94	0.30	0.21	0.07	0.99	0.59	0.74

For all noise levels considered, exceptional precision (> 0.9) was observed. While recall drops with larger levels of experimental noise, the observed recall values are nevertheless significantly higher than expected by chance (recall_{random}). We explain low recall values by the fact, that although post-transcriptional gene regulation occurs, the production model may still explain the protein expression time-course by adapting model parameters. Hence, we tested an additional dataset, in which required post-transcriptionally regulated proteins cannot be explained by adjusting parameters of the production model. Accordingly, in this approach the distance between the *fitted* production model and post-transcriptional gene regulation needed to be above the given threshold for a gene to be considered as post-transcriptionally regulated. In this scenario in which post-transcriptional gene regulation has a clear effect on the expression time-course, we can identify 99% of all post-transcriptionally regulated proteins (recall) at a precision of 0.94.

We further assessed the correlation between estimated model parameters and true parameters chosen to simulate regular translation. As a result, initial protein expression ($y0$) was estimated with exceptional high accuracy (Pearson correlation $\rho > 0.99$) regardless of the error level chosen. Also estimated production and degradation rates correlated well with their true values (Pearson correlation $\rho = 0.58 - 0.87$).

Supplementary references

[1] Graveley, B., Brooks, A. & Carlson, J. The developmental transcriptome of Drosophila melanogaster. *Nature* **471**, 473–479 (2011). URL `http://www.nature.com/nature/journal/v471/n7339/abs/nature09715.html`.

[2] Cox, J. *et al.* Maxlfq allows accurate proteome-wide label-free quantification by delayed normalization and maximal peptide ratio extraction. *Molecular & cellular proteomics* mcp–M113 (2014).

[3] Schwanhäusser, B. *et al.* Global quantification of mammalian gene expression control. *Nature* **473**, 337 (2011).
