# Supplementary material for: Quantifying post-transcriptional regulation in the development of Drosophila melanogaster
Source: Nat Commun. 2018 Nov 26;9:4970. doi: 10.1038/s41467-018-07455-9 (PMC6255845; doi:10.1038/s41467-018-07455-9)
Supplement: Supplementary file 3 — Description of Additional Supplementary Files [file 41467_2018_7455_MOESM3_ESM.pdf]

Description of Additional Supplementary Files

File Name: Supplementary Data 1
Description: Results of model fitting and protein classification applied to paired mRNA/protein data: The table contains data for 3761 mRNA/protein pairs as well as relevant results obtained from the model fitting and protein classification analysis. A detailed description of each column is given in sheet 2 of the excel file. While f1 denotes results obtained using the full dataset, f2 indicates results obtained from analyzing post MZT (3h-20h) data only.
