# Supplementary material for: Quantifying post-transcriptional regulation in the development of Drosophila melanogaster
Source: Nat Commun. 2018 Nov 26;9:4970. doi: 10.1038/s41467-018-07455-9 (PMC6255845; doi:10.1038/s41467-018-07455-9)
Supplement: Supplementary file 5 — Reporting Summary [file 41467_2018_7455_MOESM5_ESM.pdf]

nature research

Corresponding author(s): Falk Butter
Stefan Legewie

Reporting Summary

Nature Research wishes to improve the reproducibility of the work that we publish. This form provides structure for consistency and transparency in reporting. For further information on Nature Research policies, see Authors & Referees and the Editorial Policy Checklist.

Statistical parameters

When statistical analyses are reported, confirm that the following items are present in the relevant location (e.g. figure legend, table legend, main text, or Methods section).

n/a	Confirmed	
☐	☒	The exact sample size (*n*) for each experimental group/condition, given as a discrete number and unit of measurement
☐	☒	An indication of whether measurements were taken from distinct samples or whether the same sample was measured repeatedly
☐	☒	The statistical test(s) used AND whether they are one- or two-sided<br>*Only common tests should be described solely by name; describe more complex techniques in the Methods section.*
☐	☒	A description of all covariates tested
☐	☒	A description of any assumptions or corrections, such as tests of normality and adjustment for multiple comparisons
☐	☒	A full description of the statistics including central tendency (e.g. means) or other basic estimates (e.g. regression coefficient) AND variation (e.g. standard deviation) or associated estimates of uncertainty (e.g. confidence intervals)
☐	☒	For null hypothesis testing, the test statistic (e.g. *F*, *t*, *r*) with confidence intervals, effect sizes, degrees of freedom and *P* value noted<br>*Give P values as exact values whenever suitable.*
☐	☒	For Bayesian analysis, information on the choice of priors and Markov chain Monte Carlo settings
☐	☒	For hierarchical and complex designs, identification of the appropriate level for tests and full reporting of outcomes
☐	☒	Estimates of effect sizes (e.g. Cohen's *d*, Pearson's *r*), indicating how they were calculated
☐	☒	Clearly defined error bars<br>*State explicitly what error bars represent (e.g. SD, SE, CI)*

Our web collection on statistics for biologists may be useful.

Software and code

Policy information about availability of computer code

Data collection

Embryo timecourse:
Readmapping (SS): STAR v2.4.0h, ensembl 79
RNAseq QC (SS): FastQC, dupRadar (Bioconductor 3.4)
Read counting (SS): htseqcount (0.9.1)
Quantification isoform abundance (SS): MISO (0.5.3)
LFQ quantification (MD): MaxQuant v1.5.2.8
iBAQ quantification (MD): MaxQuant v1.5.2.8

Hrb98DE knock-down in vivo:
LFQ quantification (MD): MaxQuant v1.5.2.8

Hrb98DE knock-down S2R+:
Readmapping (SS): STAR 2.5.1b, ensembl 90
RNAseq QC (SS): FastQC, dupRadar (Bioconductor 3.4)
Readcounting (SS): FeatureCounts (Subread v1.4.6-p2)
LFQ quantification (MD): MaxQuant v1.5.2.8

Data analysis

Embryo timecourse:
Data imputation (MD): logspline R v2.1.9, zoo R package v1.8.0
Mapping FBpp -> FBtr -> Fbgn (KB): FlyBase File 2015 03
Hierarchical clustering (KB): scipy.cluster (scipy v0.17.0)
Correlation analysis (KB): scipy.stats (scipy v0.17.0)
Protein classification (KB):
custom code (https://github.com/Legewie/PyProt -DOI: 10.5281/zenodo.1435819)
scipy.optimize (scipy v0.17.0),
pyDOE.lhs (pyDOE v0.3.7),
statsmodels (statsmodels v0.5.0)

GO term analysis (KB): GOrilla, rpy2 (v2.5.6), GoSemSim (v2.6.2)
Motif analysis (KB): AME (version 4.11.2)

Hrb98DE knock-down in vivo:
Mapping FBpp -> FBtr -> Fbgn (KB): FlyBase File 2017_02
Statistics proteome (KB): scipy.stats (scipy v0.17.0)

Hrb98DE knock-down S2R+:
Statistics mRNA (SS): DESeq2 (v1.14.1)
Statistics splicing (SS): DEXSeq (Bioconductor 3.4)
Mapping FBpp -> FBtr -> Fbgn (KB): FlyBase File 2018_03
Statistics proteome (KB): scipy.stats (scipy v0.17.0)
GO term analysis (KB): scipy.stats (scipy v0.17.0), FlyMine v45.1 2017

For manuscripts utilizing custom algorithms or software that are central to the research but not yet described in published literature, software must be made available to editors/reviewers upon request. We strongly encourage code deposition in a community repository (e.g. GitHub). See the Nature Research guidelines for submitting code & software for further information.

Data

Policy information about availability of data

All manuscripts must include a data availability statement. This statement should provide the following information, where applicable:

- Accession codes, unique identifiers, or web links for publicly available datasets
- A list of figures that have associated raw data
- A description of any restrictions on data availability

The mass spectrometry proteomics data have been deposited to the ProteomeXchange Consortium via the PRIDE partner repository with the dataset identifier PXD011238 [http://proteomecentral.proteomexchange.org/cgi/GetDataset?ID=PXD011238]. The RNA-Seq data of this publication have been deposited in NCBI's Gene Expression Omnibus and are accessible through GEO Series accession numbers GSE121160 [https://www.ncbi.nlm.nih.gov/geo/query/acc.cgi?acc=GSE121160] and GSE121161 [https://www.ncbi.nlm.nih.gov/geo/query/acc.cgi?acc=GSE121161].

Field-specific reporting

Please select the best fit for your research. If you are not sure, read the appropriate sections before making your selection.

☒ Life sciences ☐ Behavioural & social sciences ☐ Ecological, evolutionary & environmental sciences

For a reference copy of the document with all sections, see nature.com/authors/policies/ReportingSummary-flat.pdf

Life sciences study design

All studies must disclose on these points even when the disclosure is negative.

Sample size

Embryonic time-course (Mass-Spectrometry+RNAseq): Proteome and transcriptome were measured at 14 time points during Drosophila embryogenesis (0h, 1h, 2h, 3h, 4h, 5h, 6h, 8h, 10h, 12h, 14h, 16h, 18h, 20h) in biological quadruplicates.

Hrb98DE knock-down in vivo (Mass-Spectrometry): Experimental knock-down was performed using two different driver-lines (actin and nanos), each with three distinct dsRNAs (in biological triplicates) and compared to control flies.

Hrb98DE knock-down in S2R+ cells (Mass-Spectrometry+RNAseq): Experimental knock-down was performed using LacZ control and dsRNA treated cells. For the transcriptome each experimental condition was performed in biological triplicates. For the proteome each experimental condition was performed in biological quadruplicates. In order to exclude off-target effects, the transcriptome measurement was repeated using an additional dsRNA. In this follow-up experiment, all experimental conditions (LacZ control, dsRNA1, dsRNA2) were performed in biological triplicates.

Data exclusions

Embryonic time-course (Mass-Spectrometry+RNAseq): For each sample, ProteinGroups needed to be quantified in at least two out of four biological replicates. Only ProteinGroups quantified at 10 out of 14 time points were included in this study. In the RNAseq only transcripts for which protein was uniquely assigned were used in this study.

Hrb98DE knock-down in vivo (Mass-Spectrometry): Only data from actin KD1 and actin KD 3 were used, as the other experimental conditions showed insufficient knock-down efficiency. Only proteins quantified in all 9 measured samples of the in vivo knock-down experiment were considered in this study. In addition only proteins overlapping with the embryonic time-course were considered.

Hrb98DE knock-down in S2R+ cells (Mass-Spectrometry+RNAseq): Transcripts falling below the expression threshold of 1.4 RPKM in any of the measured samples were removed. Only ProteinGroups with uniquely assigned gene identifier (FBgn) were considered in the study. Only ProteinGroups quantified in all samples (lacZ control x 4, dsRNA x 4) were considered.

Replication	Hrb98DE knock-down in S2R+ cells (RNAseq): In order to exclude off-target effects, the transcriptome measurement was repeated using an additional dsRNA.
Randomization	No randomization was performed in this study.
Blinding	No blinding was performed in this study.

Reporting for specific materials, systems and methods

Materials & experimental systems

n/a	Involved in the study
☒	☐ Unique biological materials
☒	☐ Antibodies
☐	☒ Eukaryotic cell lines
☒	☐ Palaeontology
☐	☒ Animals and other organisms
☒	☐ Human research participants

Methods

n/a	Involved in the study
☒	☐ ChIP-seq
☒	☐ Flow cytometry
☒	☐ MRI-based neuroimaging

Eukaryotic cell lines

Policy information about cell lines

Cell line source(s)	Hrb98DE knock-down in S2R+ cells: DGRC, stock #150 (https://dgrc.bio.indiana.edu/)
Authentication	DGRC, stock #150
Mycoplasma contamination	No evidence of contamination (RNA-Seq)
Commonly misidentified lines (See ICLAC register)	-

Animals and other organisms

Policy information about studies involving animals; ARRIVE guidelines recommended for reporting animal research

Laboratory animals	Drosophila melanogaster (wild-type Oregon R), Bloomington Drosophila Stock Center #31303 and #32351
Wild animals	The study did not involve wild animals.
Field-collected samples	The study did not involve samples collected from the field.
